# Time Course of Metabolomic Alterations in Cerebrospinal Fluid After Aneurysmal Subarachnoid Hemorrhage

**DOI:** 10.3389/fneur.2020.00589

**Published:** 2020-06-23

**Authors:** Wing Mann Ho, Alice S. Görke, Bernhard Glodny, Herbert Oberacher, Raimund Helbok, Claudius Thomé, Ondra Petr

**Affiliations:** ^1^Department of Neurosurgery, Medical University Innsbruck, Innsbruck, Austria; ^2^Department of Radiology, Medical University Innsbruck, Innsbruck, Austria; ^3^Department of Forensic Medicine, Medical University Innsbruck, Innsbruck, Austria; ^4^Department of Neurology, Medical University Innsbruck, Innsbruck, Austria

**Keywords:** metabolomics, subarachnoid hemorrhage, early brain injury, cerebrospinal fluid, metabolites, biomarker, delayed cerebral ischemia

## Abstract

**Object:** The aim of this study was to investigate metabolite levels in cerebrospinal fluid (CSF) in their time-dependent course after aneurysmal subarachnoid hemorrhage (aSAH) comparing them to patients harboring unruptured intracranial aneurysms.

**Methods:** Eighty CSF samples of 16 patients were analyzed. The study population included patients undergoing endovascular/microsurgical treatment of ruptured intracranial aneurysms (*n* = 8), which were assessed for 9 days after aSAH. Control samples were collected from the basal cisterns in elective aneurysm surgery (*n* = 8). The CSF samples were consecutively collected with extraventricular drain (EVD) placement/intraoperatively, 6 h later, and daily thereafter (day 1–9). The endogenous metabolites were analyzed with a targeted quantitative and quality controlled metabolomics approach using the AbsoluteIDQ®p180Kit. Differences inbetween timepoints and compared to the control group were evaluated.

**Results:** Numerous alterations of amino acid (AA) levels were detected within the first hours after bleeding. The highest mean concentrations occurred 1 week after aSAH. AA levels were continuously increasing over time starting 6 h after aSAH. Taurine concentration was highest briefly after aSAH starting to decrease already after 6 h (vs. day 1–9, *p* = 0.02). The levels of sphingomyelins/ phosphatidylcholines/ lysophosphatidylcholines/mono-unsaturated fatty acid chain were highly elevated on day 0 (compared to other timepoints or controls, *p* < 0.01) and decreased over the next several days to concentrations comparable to the control group. Carnitine concentrations were decreased after SAH (vs. day 7, *p* < 0.01), while they recovered within the next day. The Fischer ratio of branched-chain AA to aromatic AA was lowest immediately after SAH and increased in 7 days (*p* < 0.001).

**Conclusion:** AA levels in CSF increased overtime and often differ from patients without SAH. There was a peak concentration of structural AA within the first 6 h after aneurysm treatment. Time-dependent alterations of CSF metabolites and compounds may elucidate pathophysiological processes after aSAH, providing potential predictors assessed non-invasively by routine lab testing.

## Introduction

Early brain injury (EBI) occurs within the first 72 h after aneurysmal subarachnoid hemorrhage (aSAH) most likely related to acute changes of intracranial pressure, cerebral perfusion pressure, and cerebral blood circulation. Notably, cellular hypoxia, energy depletion, neuronal stress, oxidative stress, free radicals, excitotoxicity, impaired protein synthesis, and DNA damage are involved in the molecular mechanism of brain injury after aSAH (1). Additionally, delayed cerebral ischemia (DCI) and cerebral vasospasm are suggested to be linked to EBI due to common pathophysiological pathways and direct interactions.

Over the last few decades many clinical trials in neurocritical care have been conducted to elucidate the pathophysiology of cerebral vasospasm and brain injury (2–4). Cerebral microdialysis enables sampling and collecting small-molecular-weight substances from the interstitial space, which has shown promising results in detecting biochemical changes in brain tissue associated with brain injury (5). Levels of lactate, pyruvate, and glucose, and glutamate (Glu) were detected as indicators of brain ischemia (6). However, the distribution and worldwide use of cerebral microdialysis as a monitoring device remains limited (5).

With continuing improvements in analytical technology, metabolomics is a promising innovative method, which examines various compounds in body fluids. Metabolomics involves the global analysis of small molecule metabolites (<1,500 Da) existing in living organisms. Applying metabolomics in many fields has led to the discovery of numerous useful biomarkers and the development of diverse and improved screening assays (7). Most frequently investigated metabolites include the excitatory neurotransmitter glutamate as well as histidine that is as an essential amino acid and precursor to histamine with strong free radical scavenging characteristics (8). Especially specific phosphatidylcholines (PC) have been shown to be putative biomarkers specific for Alzheimer's disease (9).

Despite considerable success in brain research (10), metabolomics has only found limited applicability for studying aSAH-related changes in cerebrospinal fluid (CSF). So far, four studies analyzing amino acid levels in CSF after aSAH have been published (11, 12). In 1985, increased levels of amino acids in CSF after aSAH were detected (11). Jung et al. (13) examined the CSF levels of glutamine, serine, and histidine. Sokół et al. (12) recently reported 33 compounds which were assayed for their study. The latest study by Lu et al. (14) used untargeted metabolomic profiling of 97 metabolites to analyze CSF samples at two time points after aSAH (admission and the anticipated vasospasm timeframe).

The aim of this study is the comprehensive investigation of metabolomics in CSF and their time-dependent alterations in the early phase after aSAH and the comparison with CSF of patients harboring unruptured intracranial aneurysms. Furthermore, the role of altered levels of inhibitory, excitatory, and structural amino acids and related compounds in the process of brain injury following aSAH shall be elucidated. To our knowledge, this is the first study investigating continuous alterations of AA levels in CSF of aSAH patients within 6 h and daily thereafter.

## Materials and Methods

This study was conducted in a single medical center in accordance with the Declaration of Helsinki, in a prospective observational manner. The study protocol and consent forms were approved by the local ethics committee.

Eighty CSF samples of 16 patients were analyzed. The study population included patients undergoing early (<24 h after SAH) endovascular or microsurgical treatment of ruptured intracranial aneurysms where an emergency extraventricular drainage (EVD) was indicated due to acute post-hemorrhagic hydrocephalus (modified Fisher grade 3) (*n* = 8). Control samples were collected during elective surgical aneurysm treatment (*n* = 8). Patients with arteriovenous malformations, fistulas, or a history of trauma were excluded. Patients aged below 18 years were not enrolled.

The clinical status of the patients on admission was assessed using the Glasgow Coma Scale (GCS). Following the standard operating procedure at our institution, EVD was inserted in patients with a GCS below 15 and (1) a relative bicaudate index >1 (2), a focal dilatation of the ventricular system due to obstruction, or (3) intraventricular blood clot. As a standard of care, screening for EVD infection was obtained at least twice a week by assessing CSF cell count and CSF culture. The EVD was inserted within 24 h after bleeding and before endovascular treatment or during microsurgical clipping.

The CSF samples were consecutively collected before aneurysm treatment (at EVD placement), during intraoperatively, 6 h later, 12 h after the intervention, and daily thereafter from day 1 till 10. The samples were forthwith centrifuged at 4°C, 1,000 rpm for 5 min (centrifuge 5810 R Eppendorf, Germany), and the supernatants were stored at −80°C until further assessment.

The endogenous metabolites were analyzed with a targeted quantitative and quality controlled metabolomics approach using the Absolute*IDQ*^®^ p180 Kit (BIOCRATES Life Science AG, Innsbruck, Austria). This validated assay allows for the comprehensive identification and the quantification of 186 endogenous metabolites. A targeted analysis for these metabolites was performed by applying flow injection analysis-tandem mass spectrometry (FIA-MS/MS) as well as liquid-chromatography-tandem mass spectrometry (LC-MS/MS) with multiple reaction monitoring in positive electrospray ionization mode. Quantification was achieved with internal standards.

The Absolute*IDQ*^®^ p180 Kit, a 96-well-plate format assay, was applied as described in the manufacturer's instructions. In short, two times 15 μl sample mixture were pipetted onto filter spots suspended in the wells of a 96-well-filter plate. The filter plate was fixed on top of a deep-well-plate serving as a receiving plate for the extract later on, i.e., a combi-plate structure. After brief drying under nitrogen stream, 50 μl of a 5% phenylisothiocyanate solution was added to enable derivatization of amino acids. After 20 min of shaking and nitrogen drying, 300 μl of 5 mM ammonium acetate in methanol was added to the wells. After 30 min of incubation, the combi-plate was centrifuged to gain the extracts into the lower receiving deep-well-plate, which was then detached from the upper filter plate. Samples for FIA-MS/MS were prepared by mixing 10 μL of the eluates with 40 μL of 5 mM ammonium acetate in methanol. For LC-MS/MS analysis, 30 mL of the eluates were diluted with 30 μl of water.

Quantitative analysis was performed on a mass spectrometric system consisting of a 1100 series HPLC pump (Agilent, Waldbronn, Germany), a CTC-PAL autosampler (CTC Analytics AG, Zwingen, Switzerland) and a QTrap 4000 mass spectrometer (Sciex, Toronto, Canada). The injection volume was 20 μl. For FIA-MS/MS, the flow rate was set to 30 μl/min. For LC-MS/MS analysis, separations were accomplished on an Eclipse XBD-C18 column (3.5 μm, 3.0 × 100 mm, Agilent) using a 5-min gradient of 0–95% acetonitrile in aqueous 0.2% formic acid solution. The flow rate was set to 500 μl/min, and the column temperature was held at 50°C. Metabolite concentrations (μM) were automatically calculated by the MetIDQ software package part of the Absolute*IDQ*^®^ p180 Kit. After quality control, quantitative results of 90 metabolites were submitted to statistical analysis.

The results are presented as mean values ± standard deviation (SD). Statistical analysis of the data was performed as suited using analysis of variance (ANOVA) followed by Tukey's test for *post-hoc* comparisons of mean values or Mann–Whitney *U*-test and *post-hoc* Benjamini–Hochberg procedure. Statistical significance was defined as *p* < 0.05.

## Results

We have applied a validated mass spectrometric assay to target 186 metabolites in CSF samples from patients and controls. Quantitative information was obtained for 40 acylcarnitines (Cx:y), hydroxylacylcarnitines [C(OH)x:y], dicarboxylacylcarnitines (Cx:y-DC), the sum of the hexoses (H1), 40 amino acids and biogenic amines, 15 sphingomyelins (SMx:y), and hydroxysphingomyelin (SM(OH)x:y), 76 phosphatidylcholines (PC aa = diacyl x:y, PC ae = acyl-alkyl x:y), and 14 lysophosphatidylcholines (lysoPC a x:y). The quantitative data was further used to calculate ratio and sum parameters that are known to be indicative for certain metabolic changes or states. Concentrations as well as derived parameters were submitted to statistical analysis.

No significant changes could be found in the time courses of creatinine, glutamate, hexoses, putrescine, symmetric dimethylarginine (SDMA), spermine, and the total dimethylarginine (DMA) levels.

The ratios of kynurenine/tryptophane, ornithine/arginine, asymmetric DMA (ADMA)/arginine, spermine/putrescine, tyrosine/phenylalanine also remained constant over time. Of note, the aforementioned metabolites showed no significant differences when compared to the CSF metabolites from the control group of patients harboring an unruptured intracranial aneurysm.

Taurine was decreased 24 h after aSAH and was significantly lower than in the CSF of patients undergoing elective aneurysm clipping, but did not differ at any other time points. The creatinine and glutamate levels were lower after aSAH at all times when compared to the control group.

The universal metabolite citrulline showed a tendency to increase on day 8, but the ratio citrulline/arginine was attenuated 12 h after treatment till day 7, as well as the decreased ratio of citrulline/ornithine till day 3.

For the following 17 AA substantially increased levels were observed reaching the maximum 1 week after aSAH: arginine, ADMA, asparagine, alanine, glutamine, histidine, isoleucine, leucine, kynurenine, lysine, methionine, proline, serine, tryptophan, threonine, tyrosine, valine. Elevation was significant and similar in all of 17 AA during the entire evaluated time course.

Divided in their subgroups the highest mean was at 1 week for branched chain AA (BCAA), essential and non-essential AA, and glucogenic AA.

The Fischer ratio (branched chain amino acids/aromatic amino acid; a predictor for extracerebral disease) was decreased after aSAH, and regenerated to control values within 6 days.

Few phosphatidylcholines showed no changes in a time-dependent manner, while most of the cholines significantly increased briefly after bleeding when compared to controls, with a subsequent decrease, usually after 6 h or later (Supplementary Table 1). Hydroxysphingomyelins, lysophosphatidylcholines, and sphingomyelins showed temporarily increased levels immediately after aSAH with an ensuing decrease to CSF-concentrations within the first 6 h that are comparable with the control group.

PCs with Monounsaturated glycerophosphocolines (MUFA-PC) demonstrated a peak level only at the time of aSAH, while the ratio of PCs with Monounsaturated and Polyunsaturated glycerophosphocolines (MUFA-PC/PUFA-PC) decreased directly after aSAH, and regenerated within the first 24 h to CSF levels of the control group. When comparing with Saturated glycerophosphocoline PCs (SFA), similar time courses were observed in MUFA/SFA and PUFA/SFA ratios. Both ratios reached their peaks instantly after aSAH, with a slow-paced decrease within the next 48 h to the control levels.

Oppositely, in terms of time acylcarnitines including C0, C2, C3, and C4 slowly increased and reached the peak on day 7. Significantly elevated levels were detected 5 days after aSAH. Unlike, the C0 and C2 levels were decreased in comparison to the control group within the first 24 h after aSAH.

## Discussion

In the early stage of aSAH, we have found several constitutive alterations of metabolite levels in the CSF. Concentrations of AA, biogenic amines as well as acylcarnitines significantly increased over time, while the majority of analyzed phosphatidylcholines, lysophosphatidylcholines, and sphingomyelins decreased substantially after aSAH.

In summary, we have detected a time-dependent elevation in the levels of amino acid concentrations in the CSF of patients suffering aSAH. To the best of our knowledge, this is the first study on the time course of CSF metabolomics in the early phase after aSAH compared to patients undergoing elective aneurysm treatment. So far, the following four papers analyzed AA in CSF in patients with aSAH (11–14).

Jung et al. (13) examined the CSF samples of 18 aSAH patients and compared them to five control samples with heterogenic indications for EVD placement. The results were pooled in 3 day time-periods for analysis. The CSF levels of glutamine, serine, and histidine were significantly increased after aSAH (13).

**Figure 1:**
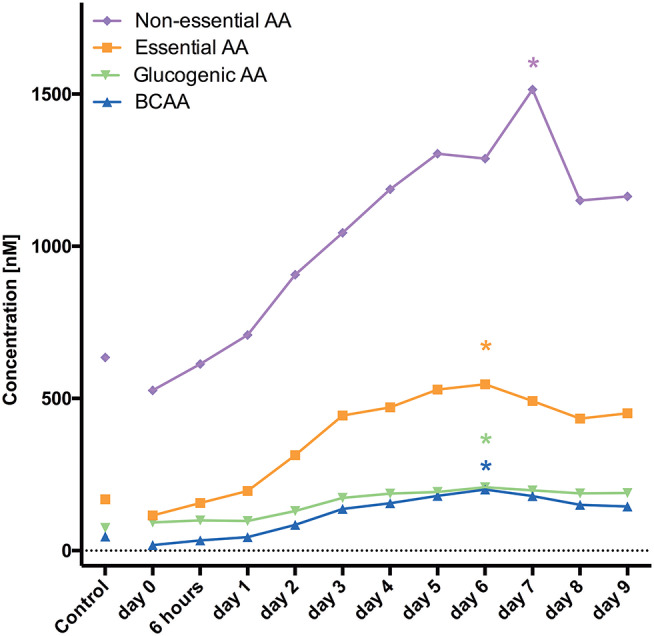
Demonstrates changes of AA levels and how they increase over time. *marks the PEAK timepoint with significant difference to the control group and timepoint 0 (*p* < 0.05).

Sokół et al. (12) analyzed CSF concentrations of 33 AA in 23 patients after aSAH. CSF samples were collected on days 0–3, 5, and 10. Asparagine, glutamine, taurine, citrulline, GABA, 3-methyl-histidine, ornithine, cystathionine, and isoleucine were elevated within the first 3 days (12). Our findings are in accordance with the latter study showing the highest mean concentrations 1 week after aSAH (Figure 1). Furthermore, AA levels were continuously increasing over time starting very early (only 6 h) after aSAH.

In the recent publication by Lu et al. (14) in 2018, untargeted metabolomic profiling was used to analyze 97 metabolites in the CSF of 15 aSAH patients at time of admission. The second time of CSF sampling was at the anticipated vasospasm timeframe (average of post-bleed day 6) or earlier if vasospasm occurred confirmed by CT or catheter angiography. Over this variable timeframe, the authors could show that 16 metabolites, primarily free amino acids, significantly changed between the two time points (14).

Taurine has proven to be neuroprotective against ischemic stroke and experimental aSAH to prevent mitochondrial dysfunction and to protect against endoplasmic reticulum (ER) stress (15–17). After traumatic brain injury (TBI), the CSF taurine concentration has been observed to be increased and returned to control value ~67 h after injury (18). Sokół et al. (12) observed increased taurine levels on day 0–3 in the CSF of aSAH patients with poor outcome. Confirming these results, we found the concentration to be highest briefly after aSAH, while starting to decrease already 6 h after EVD placement. Importantly, the highest taurine level in our prospective study did not differ from patients undergoing elective aneurysm clipping.

Sphingomyelin is found in cell membranes, especially in the membranous myelin sheath that surrounds nerve cell axons. It has been shown to be a myelin biomarker in CSF of acquired demyelinating neuropathies. As in acquired demyelinating peripheral neuropathies, myelin breakdown occurs, the sphingomyelin amount in CSF of these patients increases, detecting myelin loss (19). Phosphatidylcholines are the major component of biological membranes and have been demonstrated to be increased in CSF samples of patients after TBI (20, 21). The highest mean concentration of lysophosphatidylcholine has been observed on day 1 after injury. Emmerich et al. (20) found the peak concentrations of phosphatidylethanolamine, phosphatidylcholine, and sphingomyelin on day 4 after TBI. In our prospective observational study, we found that the levels of sphingomyelins, phosphatidylcholines, and lysophosphatidylcholines in the CSF were highly elevated on the day of aSAH (day 0). Afterwards, they decreased over the next several days to concentrations comparable to the control group. We suggest that an increased lipid concentration in the CSF indicates early brain injury, amount of cell death, and the degradation products after aSAH.

Acylcarnititine and carnitine act as transporters of long-chain fatty acids into the mitochondria to be oxidized and produce energy via ß-oxidation. In *in-vitro* and *in-vivo* studies of neuronal death, acylcarnitine showed neuroprotective effects (22). Three separate studies using a clinically relevant canine cardiac arrest and resuscitation model, have demonstrated that immediate post-ischemic administration of acetylcarnitine prevented free radical-mediated protein oxidation in the frontal cortex (23–25). We discovered significantly decreased carnitine concentrations in CSF early after aSAH, while it recovered within the next several days to control levels. It can be speculated that aSAH triggers a higher energy metabolism due to ischemic stress. Therefore, it leads to migration of carnitine decreasing the CSF levels at the beginning. Reestablishing normal concentrations may be due to a lower demand over time, and/or due to an increased carnitine synthesis.

The Fischer ratio (branched chain amino acids/aromatic amino acids) in plasma has shown a predictive value regarding the risk of metabolic disease (26), hepatic encephalopathy (27), and pulmonary hypertension (28). With decreased plasma levels of BCAA and thus a low ratio of BCAA/aromatic AA the toxic aromatic AA are allowed to penetrate the blood-brain barrier which leads to hepatic encephalopathy (27). In patients with pulmonary hypertension, the Fischer ratio decreases in proportion to the clinical severity of the disease (25).

In our prospective study, the Fischer ratio was lowest right after aSAH occurred. It completely regenerated thereafter within 7 days, and was then similar to the control group. This observation may represent an autoregulation in the AA homeostasis, with balance and recovery after aSAH. Assuming the functional compensating mechanisms, altered levels of AA can result in an elevated regeneration activity demanding more AA after aSAH, therefore leading to lower AA levels in the early phase. The increasing concentrations over time may be due to elevated peptidase activity and peptide degradation, and/or increased release.

To evaluate the relevant pathophysiologic mechanisms of aSAH and its related morbidity/mortality further detailed investigation including the proteomics and metabolomics relations are required. The role of AA alterations associated with aSAH pathophysiology and clinical setting will need to be investigated in further studies.

## Conclusion

Changes of restricted markers in cerebral metabolism are detectable with microanalysis catheters, yet routinely used biomarkers in CSF after aSAH are still lacking. In this prospective observational study numerous significant consecutive in-depth alterations of various metabolites (AA) concentrations in the CSF in the time course after aSAH could be demonstrated. These are detectable by metabolomics after aSAH and often differ from controls. There was a peak of structural AA within the first 6 h after EVD placement.

Evaluating the CSF metabolites and compounds, and their time-dependent alterations, may elucidate pathophysiological processes after aSAH and may provide new predictive biomarkers that can be evaluated with laboratory routine without additional invasive methods.

## Data Availability Statement

The datasets generated for this study are available on request to the corresponding author.

## Ethics Statement

The studies involving human participants were reviewed and approved by Ethic committee Medical University Innsbruck. The patients/participants provided their written informed consent to participate in this study.

## Author Contributions

WH: acquisition and analysis of data and data interpretation. AG: acquisition and interpretation of data. BG: statistical analysis of data and data interpretation. HO: chemical analysis, data interpretation, and revisions. CT: design of the study and revisions. RH: analysis and interpretation of data and revisions. OP: conception/design of the study, acquisition and analysis of data, data interpretation, and revisions. All authors: approved the submitted version and have agreed both to be personally accountable for the author's own contributions and to ensure that questions related to the accuracy or integrity of any part of the work, even ones in which the author was not personally involved, have been appropriately investigated, resolved, and the resolution documented in the literature.

## Conflict of Interest

The authors declare that the research was conducted in the absence of any commercial or financial relationships that could be construed as a potential conflict of interest.
