# Supplementary material for: Time Course of Metabolomic Alterations in Cerebrospinal Fluid After Aneurysmal Subarachnoid Hemorrhage
Source: Front Neurol. 2020 Jun 23;11:589. doi: 10.3389/fneur.2020.00589 (PMC7324721; doi:10.3389/fneur.2020.00589)
Supplement: Supplementary file 1 [file Table_1.docx]

Supplementary Table 1: Mean and SD values of selected metabolites

|  |  | Control | day 0 | 6 hours | day 1 | day 2 | day 3 | day 4 | day 5 | day 6 | day 7 | day 8 | day 9 |
| --- | --- | --- | --- | --- | --- | --- | --- | --- | --- | --- | --- | --- | --- |
| essential AA | Mean | 168,800 | 115,500 | 156,200 | 196,400 | 313,600 | 443,900 | 470,800 | 529,200 | 546,600 | 491,400 | 433,700 | 451,000 |
|  | SD | 109,300 | 29,890 | 86,820 | 76,480 | 97,400 | 187,100 | 197,800 | 145,500 | 389,000 | 324,300 | 340,700 | 326,300 |
| non-essential | Mean | 634,300 | 526,200 | 613,300 | 708,800 | 906,400 | 1044,000 | 1187,000 | 1304,000 | 1288,000 | 1515,000 | 1150,000 | 1164,000 |
|  | SD | 100,500 | 47,350 | 146,100 | 136,800 | 148,800 | 229,400 | 249,000 | 241,500 | 475,800 | 769,300 | 511,700 | 527,900 |
| BCAA | Mean | 46,330 | 18,160 | 33,400 | 44,060 | 84,560 | 136,700 | 155,900 | 179,900 | 200,500 | 179,200 | 150,200 | 145,300 |
|  | SD | 60,890 | 7,674 | 33,470 | 21,720 | 35,990 | 76,790 | 95,420 | 62,790 | 191,600 | 164,300 | 170,200 | 136,200 |
| glucogenic AA | Mean | 74,880 | 92,570 | 99,330 | 97,480 | 130,500 | 173,200 | 187,700 | 192,600 | 208,000 | 197,800 | 187,800 | 189,600 |
|  | SD | 26,890 | 10,300 | 34,390 | 25,830 | 25,250 | 45,200 | 47,040 | 33,220 | 121,000 | 78,630 | 91,570 | 82,760 |
| Fischer Ratio | Mean | 0,977 | 0,459 | 0,568 | 0,689 | 0,823 | 0,958 | 1,034 | 1,077 | 1,128 | 1,277 | 1,053 | 0,937 |
|  | SD | 0,458 | 0,124 | 0,209 | 0,123 | 0,168 | 0,181 | 0,254 | 0,168 | 0,290 | 0,561 | 0,430 | 0,361 |
| Taurine | Mean | 18,590 | 14,940 | 10,260 | 8,243 | 8,928 | 12,220 | 11,120 | 11,150 | 14,710 | 11,180 | 10,720 | 9,400 |
|  | SD | 10,370 | 6,267 | 2,944 | 2,739 | 2,899 | 5,464 | 3,182 | 3,496 | 7,950 | 3,822 | 2,938 | 3,807 |
| ADMA | Mean | 0,092 | 0,074 | 0,101 | 0,103 | 0,117 | 0,127 | 0,173 | 0,181 | 0,187 | 0,225 | 0,186 | 0,168 |
|  | SD | 0,043 | 0,022 | 0,014 | 0,017 | 0,025 | 0,023 | 0,049 | 0,039 | 0,069 | 0,145 | 0,088 | 0,078 |
| totalDMA/Arg | Mean | 0,013 | 0,019 | 0,019 | 0,011 | 0,008 | 0,008 | 0,010 | 0,009 | 0,009 | 0,007 | 0,008 | 0,008 |
|  | SD | 0,004 | 0,003 | 0,005 | 0,003 | 0,002 | 0,003 | 0,003 | 0,002 | 0,003 | 0,003 | 0,002 | 0,003 |
| Arg | Mean | 31,650 | 17,880 | 20,530 | 25,630 | 33,110 | 39,620 | 41,010 | 43,500 | 46,330 | 44,350 | 42,970 | 43,550 |
|  | SD | 36,100 | 1,147 | 5,171 | 5,899 | 7,471 | 8,215 | 7,075 | 9,322 | 17,460 | 10,210 | 16,180 | 18,080 |
